# Psychosocial and Service Delivery Impacts of the COVID-19 Pandemic on Children With Respiratory Conditions, Their Parents and Their Health Care Providers

**DOI:** 10.1177/00469580241246338

**Published:** 2024-04-11

**Authors:** David B. Nicholas, Sherri Lynne Katz, Jill Ciesielski, Rosslynn T. Zulla

**Affiliations:** 1 Faculty of Social Work, Central and Northern Alberta Region, University of Calgary, Edmonton, AB, Canada; 2 Children’s Hospital of Eastern Ontario, Ottawa, ON, Canada; 3 University of Ottawa, Ottawa, ON, Canada; 4 Children’s Hospital of Eastern Ontario Research Institute, Ottawa, ON, Canada

**Keywords:** respiratory medicine, pediatrics, COVID-19, pandemic, psychosocial impact

## Abstract

The COVID-19 pandemic imposed widespread impacts on the health and well-being of children with respiratory challenges and their families, as well as on the health care system that supports them. An exploratory qualitative study was undertaken to examine how the pandemic impacted families’ and health care providers’ daily lives and experiences of care. Four youth, 12 parents and 7 health care providers participated in interviews via telephone or online technology. Content analysis of transcribed interviews revealed participant experiences, including initial responses to the pandemic, adjustment to pandemic shifts, and anticipation of the future. While deleterious physical health impacts were minimal for children with pre-existing respiratory conditions, their mental health was negatively impacted by the pandemic and related health protocols. Families and health care providers experienced strain, yet demonstrated resilience. Pandemic-related shifts profoundly impacted daily life at home, school, and work. Pediatric pandemic planning in clinical care is recommended to better address the needs of children with respiratory conditions and their families as well as pediatric health care providers.

**What do we already know about this topic?**
The COVID-19 pandemic has had multiple psychosocial impacts on vulnerable children and their families.
**How does your research contribute to the field?**
Findings expand knowledge about the impacts of the COVID-19 pandemic in a Canadian context, with a focus on how public health protocols and shifting clinical care affected children with respiratory conditions, their families and health care providers amidst the constrained circumstances of the COVID-19 pandemic.
**What are your research implications toward theory, practice, or policy?**
Results from this study emphasize the need to build capacity at practice and broader health program and organization levels in order to mitigate negative service delivery impacts for children with respiratory issues and their families.

## Background

As were others, individuals with respiratory conditions and their families were affected by the COVID-19 pandemic. A study addressing pandemic impacts reported that individuals 16 to 49 years of age with severe asthma were at heightened risk to require critical care, and this population experienced deleterious outcomes including mortality.^1^ In contrast, a recent scoping review reported that individuals with asthma, compared to those without asthma, had a lower risk of acquiring COVID-19, and were less likely to develop severe illness from COVID-19 or require ICU admission or mechanical ventilation.^2^ A systematic review reported that individuals with chronic respiratory conditions (eg, emphysema, chronic bronchitis), compared to individuals with asthma, were at greater risk for COVID-19 related hospitalization, ICU admission, and mortality.^3^

Current systematic reviews have primarily reflected adult populations. However of studies focusing on children, negative psychosocial outcomes (eg, anxiety, depression) have been reported among children with asthma^4^ and their parents.^5,6^ In a qualitative study, parents reported fear about their child contracting COVID-19, challenges in the family such as managing family dynamics and child care concerns during the pandemic, and service delivery changes such as increased reliance on virtual care.^6^ Parents drew on an array of supports including informal support (eg, friends and family) formal support (eg, parent support groups), and financial resources (eg, government programs offering financial benefits) to cope with these changes.^6^

Among pediatric populations, negative impacts of COVID-19 have largely reflected children’s mental health and coping. Since the onset of the pandemic, parents noted that their children with asthma who adhered to their medication protocol had better control of their asthma.^7,8^ However as the pandemic and related restrictions continued, negative psychosocial impacts were observed among pediatric respiratory care populations. Studies have reported that children with chronic respiratory conditions (eg, cystic fibrosis, asthma, tuberculosis, rhinitis allergica) experienced decreased physical activity,^7,9^ less sleep,^7^ more disagreements with family members,^6,9^ and more screen time, that is, television, computer and telephone use.^7,9^ COVID-19-related anxiety and psychiatric symptoms were reported to have predicted 27% of poor quality of life among children with asthma.^10^ Recent studies have demonstrated mid and longer-term impacts of the pandemic on the mental well-being of children and adolescents.^11,12^ Essler et al^11^ found that child and family-related well-being steadily decreased over the course of the pandemic. This finding is concerning given that a meta-analysis of studies revealed that children with chronic conditions already may be at slightly elevated risk for psychosocial distress^13^; thus, stressors emerging from the pandemic may have negatively and differentially impacted well-being and sense of vulnerability, particularly among children with respiratory conditions and/or those with pre-existing illness.

Among parental caregivers, anxiety experienced during the COVID-19 pandemic has been correlated with heightened fear about their child’s risk of contracting COVID-19 due to pre-existing illness,^7,8^ particularly when their child was prematurely born,^7^ had a history of substantial health follow-up visits,^7^ experienced prior hospitalizations,^7^ and/or had more than 1 respiratory illness.^7^ Other parent stressors during the pandemic included financial stress.^8^ Related concern reflected increased difficulty paying for a child’s medical costs and finding medications.^5,8^

Along with negative outcomes, studies conversely have suggested vicarious positive effects for some children and families. For instance, a study reported that parents benefited from home quarantine as they had additional time to attend to their child’s asthma care.^8^ Information and guidance from heath care providers via remote/digital platforms helped parents manage their child’s asthma care.^8^ Increased time at home was also beneficial to children with chronic respiratory conditions as parents observed them as more productive during the day.^9^ In a recent study, parents of children with asthma emphasized the need for tailored guidance to maintain safety (eg, means to protect asthmatic children, guidance to manage symptoms and medication) particularly early in the pandemic.^6^

Beyond these limited and mixed findings, ongoing gaps remain in the literature. There is limited understanding about how children with respiratory conditions and their families have fared over the course of the COVID-19 pandemic. Little is known about how the pandemic has impacted children specifically with respiratory conditions and their families as well as their health care providers within a Canadian context. Such information is vital to inform care guidelines and other needed supports for children with respiratory conditions and their families. To address these gaps, this study explored the lived experiences of children with respiratory conditions and their families. It also examined care impacts, and the experiences of health care providers offering respiratory care. Research questions were as follows: (i) What were the psychosocial and service delivery impacts of the COVID-19 pandemic on children with respiratory conditions and their families as well as on their health care providers?, (ii) What were the barriers and facilitators to effective health care delivery for these children during the pandemic?, and (iii) What are recommended means of optimizing pediatric respiratory care in a pandemic?

## Methods

As part of a larger study, a qualitative approach was used to elicit the experiences of participants related to research questions. This approach is well-suited for amplifying the perspectives and meanings attributed to a given phenomenon—in this case, the COVID-19 pandemic,^14^ and how respondent groups adapted and made meaning of their experience.^14^ Participants were initially sampled from a respiratory program in a central Canadian pediatric hospital. Inclusion criteria for family participants included a child under 18 years with a pre-existing respiratory condition in receipt of ongoing health care, and/or their parent(s). For health care providers, inclusion entailed the provision of pediatric respiratory medicine-based care during the pandemic. Recruitment proceeded from November, 2020 to March, 2021.

Participants were provided study information through telephone and email messages informing potential participants about the study. They were invited to engage in a 1-time qualitative interview. A semi-structured interview guide was developed by this team, and interviews were facilitated by 2 members of the research team [DBN, RTZ] through Zoom technology or by telephone. All interviews were audio recorded using an audio manual recorder. Only invited participants and the interviewer were present during interviews.

For families, questions focused on how the COVID-19 pandemic had an impact on daily experience and health care. Examples of questions are: “What has been your experience during the COVID-19 pandemic?,” “How did you cope during the pandemic?,” “Based on your experience, how do you see the future?,” and “What are some lessons learned about pediatric respiratory care in a pandemic?” Health care providers were asked questions exploring the impact of the pandemic on service delivery, as well as on professional and personal well-being. Examples of questions are: “How did the COVID-19 pandemic impact pediatric respiratory care?,” “How did the pandemic impact you professionally and personally?” and “What are suggestions for improving pediatric respiratory care during a pandemic?.” Interview duration was 0.5 to 1 h, and upon review of the dataset, it was determined that data saturation was achieved based on reflection on interview content.

Interviews were transcribed by a professional transcriber, with personal information removed to ensure anonymity. Transcripts were analyzed using a qualitative content analysis approach,^15^ given its ability to remain grounded in participants’ words and perspectives. Transcripts were analyzed using a 3-step process^15^: (1) reviewing the transcripts to generate an understanding of the data (preparation), (2) conducting an inductive analysis (organizing), and (3) creating a map of the analyzed data to generate themes (reporting). This inductive analysis approach comprised 3 steps: coding data to create meaningful units (line-by-line coding), organizing these units into a coherent schema (creating categories), and generating an interpretation of these units (determining themes).^15^ Analysis was undertaken by 1 team member [JC], with guidance and review by other team members. The analysis process was supported by NVivo 12 data management and analysis software. Emergent codes, categories and themes were discussed amongst team members for verification on a weekly basis. Final themes were shared with team members who bring expertise in qualitative research methods [DBN, RTZ] and who provide care to families of children with pre-existing respiratory conditions [SLK]. To ensure rigor, peer debriefing, referential adequacy and an audit trail^16^ were completed.

Institutional research ethics board approval was received through the University of Calgary, Conjoint Health Research Ethics Board, prior to study commencement. Participant confidentiality was ensured, and additional psychosocial support was available to participants if needed, but was not requested by any participants. All participants provided informed consent prior to study participation. Research team members involved in data collection had no prior relationship with participating family members. All team members bring to the study higher education degrees (eg, PhD) and professional designations, and they bring varying experience in pediatric health care research with senior team members [DBN, SLK] having expertise in pandemic-related pediatric research (eg, COVID-19, SARS). All team members are researchers, with 1 member [SLK] being a senior practitioner in pediatric respirology.

## Results

Twenty-three participants were interviewed, comprising 12 parents, 4 children/youth and 7 health care providers. All represented children were being treated within the local pediatric respiratory care program. Health care providers consisted of pediatric respirologists, nurses, and an allied health care provider, all of whom provided care during the pandemic.

Participants described their experience in the context of three broad thematic domains: adjusting to the pandemic, reflection and growth, and recommendations—each with sub-themes. Demographic features of the sample are outlined in Table 1. Overall, the findings identify families’ reaction to, and experience of, the pandemic which included enduring restrictions, vigilance in sanitation and safety, and limited external contact. Children/youth and parents experienced substantial educational/vocational change such as shifts and challenges in school and employment contexts. Navigating health care required new processes and resultant struggles, and families described varying physical health and psychosocial impacts including health and mental health challenges. Health care providers recalled shifts in the workplace requiring them to adapt care by transitioning to online platforms.

**Table 1.** Demographic Data (n of Represented Children/Families).

Child sex	Female (9)	Male (4)
Child age range	16 months-16 years	6-15 years
Child condition	Asthma (5); Cystic Fibrosis (1); Interstitial Lung Disease (1); Cystic Fibrosis and Asthma (1); Coughing Virus Syndrome, Global Developmental Delay, and Apnea (1)	Asthma and Allergies (3); Borderline Sleep Apnea (1)
Ethnicity	Caucasian (6); Indigenous (2); Bi-racial (1)	Caucasian (2); Bi-Racial (2)
Home region	Urban (5); Suburban (3); Rural (1)	Urban (1); Suburban (1); Rural (2)

### Initial Reaction

At the initial onset of the COVID-19 pandemic, children/youth, parents and health care providers experienced confusion, disorientation and anxiety. Parents and children/youth most commonly reported dealing with many unknowns. They described seeking information, feeling stressed and frightened, and being very cautious. Some parents expressed heightened uncertainty and worry because of their child’s pre-existing respiratory condition. They worried whether their child would continue to receive health care supports, and/or how to ease their child’s anxiety. Some were not significantly concerned at first, then realized the extent and severity of the pandemic, resulting in exponentially heightened stress. This became particularly heightened when family members perceived these children/youth to be at greater risk of contracting COVID-19 than were peers:

*I wasn’t overly concerned with the virus at first. When it got a little bigger and we had some more information on it and what it was, it did become a little scary because everybody in my household, my mother, my father, and myself were all considered high risk. Me with [a health condition], my dad who is a senior, and then my mother who just doesn’t have the best of health. It became a worry as it got bigger and we found out more about it.*

Health care providers sought to assist parents who were experiencing fear and anxiety, while simultaneously trying to manage their own emotions of fear and worry due to, for some, being at higher risk for contracting COVID-19 and working in health care. Some health care providers felt helpless because of uncertain risk particularly early in the pandemic, heightened by a lack of resources and infrastructure (eg, a lack of guidance on how to work remotely, insufficient processes for setting up virtual appointments with patients and families), and unclear pandemic-specific operational guidelines. Health care providers faced pressure to provide information to families while filtering an overwhelming amount of information, as highlighted by a health care provider:

*[Prior to the pandemic], I would probably field about ten or fifteen – rarely more – difficult issues as opposed to once COVID started, we were talking about thirty. . . [or] forty people a day asking, “Is it safe for my child?,” “What’s happening?.” Because we didn’t have that information as well because it was new to everybody. . . it was very, very hard.*

### Adjusting to the Pandemic

#### Practicing sanitation and safety

Following the initial onset of the pandemic, parents and children/youth had to adjust to the implementation of COVID-19 protocols in their care. Parents and children/youth also reported strict cleaning and safety/precautionary behaviors such as masking, sanitizing, frequent hand washing, showering and changing clothes upon entering the family home, and cleaning incoming items such as groceries. On the other hand, health care providers noted some resistance from other parents about wearing masks when in hospital, and some asked for an exemption to the protocols for their child and/or sought relaxed visitation restrictions.

#### Revising social gatherings

Many families made modifications in their social gatherings. While many largely stopped attending indoor gatherings of family or friends throughout the pandemic, most practiced social distancing if attending outdoor gatherings, and some only allowed small social circles of contact. Some families chose to implement extensive safety measures in daily life (eg, designating 1 person in the family to shop, reverting to online shopping, highly restrictive or selective visitation). Several children and youth socialized virtually through online platforms (eg, social media, texting, online gaming). However, parents and youth recalled that social distancing had negative impacts on their mental health (eg, isolation, loneliness, frustration, fear of the unknown, periodically intensified stress) and physical activity (eg, less physical exertion, limited or no access to previously enjoyed recreational activities in the community). Despite these challenges, online social engagement and recreation (eg, trying new hobbies, outdoor activities such as walking, running or skiing) were described to buffer stress.

#### Educational shifts

Transition to remote schooling resulted in multiple challenges for children and youth such as not staying focused and engaged, and feeling disconnected from teachers and peers. Parents described greater burden to advance their child’s academic progress, and they felt that their child’s learning ultimately was slowed. Unfortunately in some cases, parents and children felt that some online schoolteachers, “*don’t really get a sense of where the kids are at*,” and “*seem to be unable to operate the technology properly*.” Despite these challenges, some parents felt that online learning offered benefits such as increased parental time with children, thus availing opportunity for one-to-one engagement with their children. A few youth felt that remote learning enabled them to improve their study practices, as highlighted by a youth:

*I would say that with [the] COVID-19 pandemic, I not only learned a lot about myself, but I’ve also learned about how my brain works and how I work as an individual when it comes to the work environment, what techniques work, how I study and organize probably because it was really not good before and it’s getting better.*

#### Employment shifts

Like others in society, employment for many participating parents substantially shifted as a result of the pandemic. Many parents worked from home during the pandemic, which some found to be positive, and some less so. Several parents lost work due to their workplace being closed, or they quit work due to fear of contracting COVID-19 at work and potentially harming their child with a respiratory condition. One youth who worked in-person throughout the pandemic expressed gratitude that her workplace had instituted precautions that made her feel safer:

*My work handles things fairly well. Yeah, there’s plenty of posters and regulatory signs and we have advertisements [suggesting] staying 6 feet apart. We have regulatory arrows like directional arrows and. . .lines on the floor for when to stop at the cash [and] to stay six feet apart. There’s also a screening log. We are required to wash our hands every time we enter the department. [When] serving customers with food you always have to put gloves on first.*

Such precautions were thought to be critically important, but not consistently applied, which impeded individuals’ sense of safety in the community.

For health care providers, changes in work included adapting to new responsibilities, online platform use for care delivery, and diverse tasks. A lack of or changing information to guide families resulted in health care providers offering deeper levels of psychosocial support beyond conventional care. Shifting demands resulted for some in a decreased workload (eg, fewer patients, calls and emergency cases), while others reported substantially greater work demands than prior to the pandemic. Work shifts seemingly varied depending on one’s role/function on the interdisciplinary team. Some health care professionals (eg, senior staff) felt overwhelmed with new tasks, whereas others lacked work and, in some cases, had to seek other employment. In buffering the various changes and challenges associated with the pandemic, organizational support was identified as critically important in helping health care providers. Changes in work routines, coupled with safety protocols (eg, social distancing), resulted in feelings of isolation, as highlighted by a health care provider:

*Now everybody’s working from home so you can’t find anybody and when you try to reach them, they may or may not be around. I think [amongst] hospital staff, there’s a real sense of social isolation, and I miss that interactivity with people.*

#### Navigation of health care

The COVID-19 pandemic imposed shifting health and service delivery experiences for families. They reported primarily accessing online appointments, with in-person appointments only available when their child’s severity of condition/symptoms required more scrupulous assessment and treatment. In-person medical appointments sometimes were delayed, and parents described delays in home-based services. This lack of services in the community further impeded families’ access to care for their child (eg, respite). For those who accessed emergency care services, many recalled that their concerns were under-recognized and under-served by staff, with their child’s care needs and issues deemed to be of low priority and urgency, and some parents reporting communication challenges with some hospital staff, particularly those who were not already known.

Perspectives about virtual care were mixed. Some families liked virtual clinic appointments because they found them convenient, especially for conversation with health care providers and/or for care education, and when physical tests or assessments were not required. Despite this appreciation for virtual care, parents generally felt that this format was inferior to in-person care in terms of depth and quality of care, and resulted in more administrative work (eg, completing surveys/data collection) and relied more heavily on children’s ability to describe their issues with less physical testing or objective or physical corroboration.

Likewise, health care providers had mixed experiences with virtual care provision. They reported virtual appointments as easier and more efficient, yet identified concern related to difficulty gathering precise information in assessments, as well as technical challenges, ethical issues (eg, privacy/confidentiality risk), and potentially less clarity in communication. One participant specifically felt that the use of online formats increased health care providers’ workload, and virtual appointments developed into a practice supplemented by additional online resources (eg, websites). Irrespective of its benefits and limitations, health care providers predicted that this increased use of virtual care would likely shift practice in patient and family education even after the pandemic:

*Moving forward, this is really going to change the way we provide education just because families are also really open to it, so we’ve actually tried to change it into a positive as much as possible.*

Shifting public health protocols created significant challenges. First, some health care providers had difficulty ascertaining when in-person appointments were required. Second, extensive safety protocols had to be implemented for in-hospital care (eg, personal protective equipment [PPE] use such as masks, sanitizing, and social distancing), with multiple protocols required of the child and parent (eg, device checks, technique checks) and the health care provider (eg, frequent cleaning of examination rooms). Third, implementation challenges were observed within the hospital including varying and sometimes inconsistent levels of adherence to operational protocols, and congested entrances to the hospital. Adherence to many of these protocols reportedly was arduous for families. For instance, to comply with social distancing rules, 1 parent had to carry her child when in public spaces, which she found personally difficult:

*I have . . . very bad arthritis. So I found it very difficult because I’m not strong, you know, to lift a child*.

In another example, it became difficult for children to understand or practice required safety protocols in the hospital.

#### Health and mental health: Varied impacts of the pandemic

Some parents viewed their child’s respiratory condition to have improved or remained stable during the pandemic, whereas others felt that their child’s condition had worsened, resulting in heightened care needs. Some families expressed challenges differentiating symptoms of COVID-19 from those of asthma or allergies. With respect to children and families’ mental health, impacts varied. Children experienced anxiety, some with concerns that went beyond COVID-19 and included issues such as societal and/or environmental issues in the geopolitical context (eg, racial tensions, climate change, war). Others reported that some children generally had adjusted well, with parents attributing positive adjustment to children’s networks of formal and informal supports, prior history of positive adjustment, and vigilance in care (eg, staying home when not feeling well).

### Reflection, Anticipation, and Growth

Despite substantial challenges, the pandemic reportedly also yielded generative or positive outcomes for some families, such as increased time for family connection and reflection on what is deemed important in their life and family. Positive shifts reportedly included spending less money, slowing down, being intentional about one’s use of time, prioritizing loved ones, projecting a calm attitude, and not taking wellness for granted. Some felt that they were now healthier as a family, had gained insight about their values, and had learned more about who they can rely on. However, there were mixed feelings about the future. For instance, some participants were doubtful that COVID-19 would be eradicated in the foreseeable future, and were concerned about lingering or long-term risks and outcomes. A range of parental hope versus concern entailed varied perspectives on how their child may fare over time. Some health care providers suggested that virtual care, offered during the COVID-19 pandemic, offers promise in ongoing care provision; however, they called for needed strategies to inform and structure virtual care, including processes for reimbursing telehealth visits and guidelines for best practices relative to virtual versus in-person care.

#### Recommendations

Recommendations offered by parents focused on mitigating pandemic-related strain through improving system-level issues and care during a pandemic. Several parents advocated for greater balance in priorities of pandemic safety *and* the mental health of children and families. This was thought to entail trusting parents’ instincts and concerns, and ensuring support to families as they sought information specific to the pandemic and their child’s underlying condition. Suggestions offered at program and organizational levels included more direct COVID-19 (or other pandemic)-specific information focused in clinical subspecialties such as respiratory medicine, and at key junctures of the pandemic (eg, communication about infection risks, long-term effects, mitigative strategies), increased safety measures in schools (eg, social distancing strategies, management of class size), increased pandemic care quality in hospital, clarity of policy/guidelines, and continued monetary support to financially-strained individuals/families throughout the pandemic. Recommendations from youth about their own coping, suggested being mindful about one’s own health risk in the pandemic, increased patience and understanding about restrictions, and school-based support to students transitioning from in-person to online schooling (and vice versa).

Recommendations offered by health care providers reflected targeted capacity-building in the healthcare system. Suggestions included improved processes for the provision of PPE, streamlined and consistent messaging, clearer quarantine guidelines, easier billing protocols for physicians, increased funding for hospitals to ensure fair compensation to physicians, and increased funding for assessment and technology access in remote areas to reduce the need for families to travel long distances. This latter recommendation was noted to require resources for respiratory testing such as pulse oximetry and spirometry in distant/remote locales. Overall, innovation was sought in advancing virtual care options both within and beyond pandemic circumstances.

## Discussion

This study explored the experiences of children with respiratory conditions and their families, and health care providers supporting these families as they navigated the COVID-19 pandemic. Participants’ experiences highlighted a process of ongoing adjustment to shifts in service delivery as well as a range of psychosocial impacts. Despite variation in experience, common themes emerged. At the initial onset of the pandemic, there was substantial confusion and anxiety among families and health care providers. A lack of clarity and coherent information resulted in increased confusion about reformulated tasks and approaches to care. To lessen such disorientation in future pandemics or health emergencies, clearer communication pathways and protocols are recommended.

Families were often left on their own to discern and explain the pandemic to their child including risks and required responses. These findings are similar to other research suggesting that families had to navigate COVID-19 risk and protocols, and independently communicate this information to their child.^17^ Such findings amplify a stronger role of professional guidance to be delivered by appropriate public health representatives. Integrated child and family support between health, education and employment sectors is needed to help families navigate across relevant sectors and thus better cope in a pandemic context (eg, health care navigation, school engagement, employment/finances, activities of daily living). To increase the capacity of families throughout the pandemic, integrated support and “checking in” on an ongoing basis could better monitor and address the needs of families.

The pandemic increasingly has been described as imposing new demands on parents (eg, household tasks with fewer supports, heightened vigilance due to children’s level of vulnerability, support for anxiety, addressing children’s educational and developmental needs) that further competed with other roles (eg, employment at home). Dawes et al^18^ reported that parental roles and tasks required during the COVID-19 pandemic created feelings of invalidation and a lack of control (eg, difficulty managing one’s range of responsibilities), but also offered opportunities for greater cohesion and resiliency within some families (eg, more time for better communication). Resource challenges created knowledge and resource gaps and mental health strain for parents and their families.^18^ In the current study, parents were instrumental in their child’s academic progress and health. As such, support for parents in their multiple roles is required to help parents optimize online learning for children, and maintain parental well-being.

Important implications for health care practice are identified. Both parents and health care providers indicated a preference to continue with a mix of virtual and in-person health care even beyond the pandemic. Virtual care was shown to render service delivery more accessible and convenient. However, caution is warranted given health care provider concern related to communication issues, a lack of precision in some online procedures/processes, and technical problems associated with virtual care. Future implementation of virtual care requires greater consideration of purposes for which approaches are preferred. Further research is warranted to explore how care can be delivered in mixed formats to best meet patient and family need. Attention to the social determinants of health and issues of equity, diversity and inclusion, must be deeply considered as technology and internet access are unequally available across regions and populations.

In terms of the overall impact of the pandemic on families, some parents noted anxiety among their children. This finding is consistent with past studies in which parents observed notable changes, including children’s mood,^4,5,19,20^ activity level,^7,9^ sleep,^7^ and energy.^9^ Enhanced mental health support needs to be integrated in service delivery within a pandemic, particularly for children who are at risk for heightened acuity. Given concerning psychosocial impacts reported in recent studies, accessible mental health supports during and after a pandemic are a priority. Of note, multiple parents conveyed how helpful it was to have supportive health care providers during the pandemic. These findings illuminate the need for accessible health *and* mental health supports in which patients and their families can obtain assistance, as needed.

Despite the predominance of challenging outcomes, resiliency was demonstrated by families and health care providers. For example, families developed effective strategies to support well-being on a daily basis. Health care providers innovated new forms of care (eg, virtual care) and strategies (eg, practicing flexibility) in their professional practice. In moving forward, supports offered to families and health care providers could focus on the enhancement and recognition of their resilience and coping skills, with the aim of addressing immediate, lingering or longer-term struggles. For instance, health care providers in this study experienced periodic stressors such as a sense of helplessness, anxiety, social isolation and moral distress. In future pandemics, additional supports may be needed to optimize coping with emergent challenges. Such findings emphasize the need for proactive measures and in particular, an infrastructure of pandemic planning such as policies, structures and practices that nurture well-being in care and workforce sustainability during such adverse circumstances. For instance, supervisory and collegial support as well as ongoing targeted information-sharing were described as generative. Proactive provision for such support is advised in pandemic preparedness.

## Study Limitations and Future Research Recommendations

It is acknowledged that there was limited representation of child and youth voices, with only 2 youth being interviewed on their own, given other young people’s preference to be interviewed with their parents. Further research with children and youth about their pandemic experience is warranted. As noted in the literature, respiratory care patients with lower socioeconomic status may have experienced greater psychosocial challenges during the pandemic.^20^ Future research with diverse samples is needed, in the aim of more deeply examining relevant intersectionalities within this population. Further study is needed in determining optimal pandemic planning and recovery strategies specific to pediatric respiratory care.

Individual circumstances and supports in families likely resulted in varied experiences, hence systematically examining mediating factors would more precisely determine factors fostering outcomes. Further, research is recommended to explore the impact of human resource supports on health care providers along with means to bolster child, family and health care provider well-being post-pandemic.

## Conclusion

Notwithstanding study limitations and recommendations for further research, this study amplifies pandemic-related experiences of pediatric respiratory medicine patients, their families and their health care providers. Results indicate the importance of pandemic-related information and services for children and their families as well as support fostering the well-being of health care providers.
